# Advancing knowledge of rapid reviews: an analysis of results, conclusions and recommendations from published review articles examining rapid reviews

**DOI:** 10.1186/s13643-015-0040-4

**Published:** 2015-04-17

**Authors:** Robin M Featherstone, Donna M Dryden, Michelle Foisy, Jeanne-Marie Guise, Matthew D Mitchell, Robin A Paynter, Karen A Robinson, Craig A Umscheid, Lisa Hartling

**Affiliations:** Alberta Research Centre for Health Evidence (ARCHE), University of Alberta Evidence-based Practice Centre, Edmonton, AB T6G 1C9 Canada; Oregon Health & Science University, Portland, OR 97239 USA; ECRI Institute - Penn Medicine AHRQ EPC and the Center for Evidence-based Practice, University of Pennsylvania, Philadelphia, PA 19462-1298 USA; Scientific Resource Center, Portland VA Research Foundation, Portland, OR 97239 USA; Johns Hopkins University Evidence-based Practice Center, Baltimore, MD 21205 USA; ECRI Institute - Penn Medicine AHRQ EPC and the Center for Evidence-based Practice and the Perelman School of Medicine, University of Pennsylvania, Philadelphia, PA 19104 USA

**Keywords:** Rapid review, Review literature as topic, Health technology assessment, Systematic review, Knowledge synthesis, Evidence-based practice

## Abstract

**Background:**

Rapid review (RR) products are inherently appealing as they are intended to be less time-consuming and resource-intensive than traditional systematic reviews (SRs); however, there is concern about the rigor of methods and reliability of results. In 2013 to 2014, a workgroup comprising representatives from the Agency for Healthcare Research and Quality’s Evidence-based Practice Center Program conducted a formal evaluation of RRs. This paper summarizes results, conclusions, and recommendations from published review articles examining RRs.

**Methods:**

A systematic literature search was conducted and publications were screened independently by two reviewers. Twelve review articles about RRs were identified. One investigator extracted data about RR methods and how they compared with standard SRs. A narrative summary is presented.

**Results:**

A cross-comparison of review articles revealed the following: 1) ambiguous definitions of RRs, 2) varying timeframes to complete RRs ranging from 1 to 12 months, 3) limited scope of RR questions, and 4) significant heterogeneity between RR methods.

**Conclusions:**

RR definitions, methods, and applications vary substantially. Published review articles suggest that RRs should not be viewed as a substitute for a standard SR, although they have unique value for decision-makers. Recommendations for RR producers include transparency of methods used and the development of reporting standards.

**Electronic supplementary material:**

The online version of this article (doi:10.1186/s13643-015-0040-4) contains supplementary material, which is available to authorized users.

## Background

Rapid review (RR) products are intended to synthesize available evidence and meet the time constraints of healthcare decision-makers [1]. Systematic reviews (SRs) typically take many months, or even years, to produce [2,3], so they may not be completed in time to inform pressing decisions. As defined by the Cochrane Handbook [2], necessary elements of a SR are as follows: clearly stated objectives, pre-defined eligibility criteria for studies, an explicit and reproducible methodology, a systematic search, an assessment of the validity of the findings of the included studies, and a systematic presentation of the characteristics and findings of the included studies [2]. RRs are seen as an attractive alternative to SRs as they may use fewer resources and take less time. Health technology assessment (HTA) agencies have embraced RRs, and a 2012 survey reported that 70% of HTA agencies offer RR products alongside standard reviews [4]. While the HTA community and producers such as the Canadian Agency for Drugs and Technologies in Health (CADTH) have conducted RRs for a long time, The Cochrane Collaboration and McMaster Health Forum have also recently initiated programmes to conduct rapid reviews (https://www.cadth.ca/rapid-response-service; http://innovations.cochrane.org/response; http://mcmasterhealthforum.org/policymakers/rapid-response-program).

This analysis of review articles about RRs was conducted as part of a larger evaluation of RR products undertaken by the rapid reviews workgroup of the Agency for Healthcare Research and Quality’s Evidence-based Practice Centers (EPC) Program [5]. The workgroup investigated existing RR products and their methods of production, guidance for RR producers, and any empirical evidence regarding the validity of RRs compared with standard SRs.

To yield evidence that will inform decisions by systematic reviewers to diversify their products by offering RRs, or to adopt efficiencies that may be demonstrated by RRs, the EPC adopted the following questions to guide their investigation:

1. What are the definitions and characteristics of rapid review products produced by key organizations (for example, purpose, audience, timelines, personnel)?
2. What methodological guidance exists for the conduct of rapid reviews? What trade-offs are incurred with different methodological approaches?
3. What empiric evidence exists comparing the results of rapid reviews with systematic reviews?

The full report of the workgroup is available online [5]. Part of the investigation involved conducting a literature search to address the questions above. As a result of that larger search, the workgroup identified publications that analysed a sample of RRs. This synthesis identifies and summarizes existing review articles about RRs and reports on what these review articles tell us about RRs. By surveying existing reviews, the workgroup aimed to identify commonalities and differences in RR methods and the benefits and drawbacks to undertaking RRs. Using reviews as a unit of analysis, this synthesis provided an overview of the current RR landscape and allowed for the identification of gaps in our understanding of RRs.

## Methods

Librarians who were part of the workgroup conducted a systematic literature search of Ovid Medline, Ovid EBM Reviews, Cochrane Methodology Register, and the EPC Program’s Scientific Resource Center (SRC) Methods Library in October and November of 2013 (see Appendix for the full search strategy). A Scopus citation reference search and a grey literature search were also performed. We searched on an inclusive range of terms (for example, rapid, mini, pragmatic, targeted, focused, and brief) to describe relevant products (for example, briefs, syntheses, reviews, and assessments) to obtain a broad collection of publications about RRs. An update search of Ovid Embase was also conducted in February 2015.

At the first level of screening of titles and abstracts, references were included if they discussed RR methods, described initiatives or programmes to produce RRs, or provided empiric evidence comparing RR methods to traditional systematic review methods. At the second level of screening, records were excluded if they did not describe rapid products within the healthcare field. An additional criterion was applied to exclude references that described mini-HTAs exclusively, as mini-HTAs are often checklists that guide decision-making and not a specific method to evaluate evidence. Titles and abstracts were screened by two investigators using ABSTRACKR software (http://abstrackr.cebm.brown.edu), with disagreements resolved by a third investigator.

During the screening process, different groupings of articles emerged: papers describing RR methods; actual RR products; empiric data exploring differences in methods, results, and conclusions between RRs and standard SR methods; and review articles about RRs. This paper presents the analysis of the review articles about RRs.

Prior to full-text analysis at the second level of screening, review articles about RRs were excluded if they were not published in English and if the authors did not mention terms relating to short, focused reviews (rapid review, evidence inventory, evidence advisory, hotline response, and so on). One investigator completed second-level screening and data extraction of the review articles using 11 general questions to allow for comparison across reviews. The questions below were modified from Brassey [6]:

1. How many RRs were analysed by the review?
2. What were the objectives of the review?
3. How were RRs defined by the review?
4. How long did it take to complete the RRs included in the review?
5. What kinds of topics or questions were addressed by the RRs?
6. What search methods were used by the RRs?
7. What kinds of analyses (narrative, meta-analyses) were used in the RRs?
8. Did the RRs included in the review analyse study quality or risk of bias?
9. For what purposes were the RRs included in the review conducted?
10. What conclusions did the RRs included in the review reach?
11. Did the review reach a conclusion about how RRs differ from SRs or full HTAs?

A narrative synthesis of the review articles was conducted and focused on definitions of RRs, the questions addressed by RRs, the methods used to conduct the RRs, and the conclusions reached by RRs.

## Results

**Figure 1:**
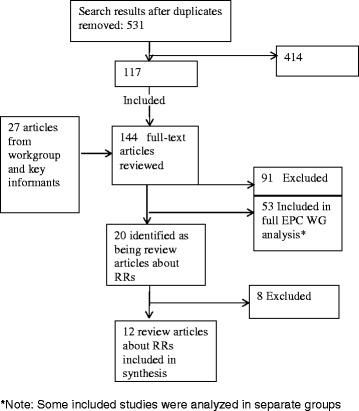
Flow of studies through the screening process.

The full-literature search yielded 531 abstracts, and 144 articles were reviewed at the full-text level. Of these, 20 articles were identified as potentially relevant review articles about RRs. Eight of those publications were subsequently excluded from the final analysis because they discussed a research method that did not fall under our broad definition of RRs [7-10], were not a review article [4,11,12], or were not published in English [13]. Twelve review articles about RRs were included in the final analysis [6,14-24] (see Figure 1 for flow of publications through the screening process). Only one included review article [15] followed methods consistent with a systematic review, as defined by the Cochrane Handbook for SRs [2].

### Review articles about rapid reviews

The 12 included review articles were published between 2003 and 2013 (see Additional file 1). The objectives of the review articles were to identify the methods used in RRs [15,16,19-23], compare RRs to SRs [6,14-18], investigate the impact of methodological differences on time to completion [16,18] and conclusions [18], examine the implications of taking methodological shortcuts compared to traditional SRs [15], describe a body of HTA products and develop a framework for using different HTA methods [23], and obtain general information about RRs [24].

RRs served as the unit of analysis for review articles except in three cases where authors also analysed methodological articles about RRs [15], product inventories [23], or survey responses from producers of rapid HTAs [24]. More than half of the review articles examined RR products conducted exclusively by HTA agencies [14,16,18,20,21,23,24]. The number of RRs examined in the review articles ranged from 2 [17] to 46 [16].

### Rapid review definitions

Authors of review articles described RRs as ‘varying widely in terms of the language used to describe them’ [15]. Other authors called RRs ‘ill-defined’ [6], ‘not well-defined’ [18], and ‘lacking a single definition’ [16]. Scott and Harstall argued that RR products ‘defy definitive categorization because of their heterogeneous timelines, components, search strategies, and methodologies’ [23]. Reviewers described rapid HTAs as falling within a continuum of assessment products, between full HTAs and mini HTAs or horizon-scanning reports [23,24]. Brassey agreed that defining RRs by a single methodology was inappropriate [6]. The 2010 SR of ‘rapid reviews’ by Ganann examined 45 methodological articles and 25 exemplars of RR methods and found many subtle differences among terms used to denote a more accelerated production process (for example, rapid, ultra rapid, succinctly timed) [15].

### Rapid review timelines

Multiple authors described rapid HTAs as taking between 1 and 6 months [14,16,18], but Dennett and Chojecki described products taking as little as 1 week [21]. Aidelsburger *et al*. reviewed rapid health-economic HTAs - defined as a targeted assessment of the cost-effectiveness of a medical technology - that took between 3 and 6 months [20]. In addition, Harker and Kleijnen’s analysis of rapid HTAs found that only 10% (*n* = 5) were published within 3 months and that the majority of reviews (51%, *n* = 25) took 7 to 12 months and another 18% (*n* = 9) took over 12 months [16].

### Rapid review scope and question types

Watt *et al*. found that the scope of RRs was narrower than that of standard SRs and that RRs were unable to address complex issues that may be of interest to decision-makers [18]. Cameron also cautioned that RRs are not quick alternatives to comprehensive SRs and that RRs should be written in response to specific questions [14].

Previous reviews described appropriate question types for RRs as being on focused topics of efficacy or effectiveness [6,14,15,17,18]. Questions considered inappropriate for RRs addressed complex interventions [6,14,15,17,18,23], economic implications [6,14,15,18,23], ethics [6,14,18,23], safety [6,18,23], and social policy [17].

RRs may also be used to answer questions about emerging technologies and to inform coverage decisions [24], to provide interim advice or ‘as a scoping mechanism for deciding when a full review is needed’ [23]. Grant and Booth emphasized the care needed to develop questions that are well-suited to a RR methodology [22].

Multiple reviews found that RR questions were narrowly focused [6,22] and that time to complete the RR was relative to the complexity of the guiding question [17]. An unexpected finding from a review article that analysed a high number of RRs was that many neglected to state a clear question. Of the 46 RRs reviewed by Harker and Kleijnen, 47% (*n* = 23) did not have a clear question, and only one RR used the Patient, Intervention, Comparison, Outcomes (PICO) criteria to formulate their question [16].

### Search and selection methods

Harker and Kleijnen found heterogeneity in search methods used in RRs [16]. Search strategies were included in 69% (*n* = 34) of the RRs, with 67% (*n* = 33) having searched the Cochrane-recommended combination of Medline, Embase, and CENTRAL databases [16]. The minimum number of databases searched was two, and the maximum number could not be determined [16]. Cameron’s review of 36 RR products found that 56% reported restricting database searching, and only 25% did any hand-searching [14]. Dennett and Chojecki’s survey of 16 producers found that 83% searched fewer databases than they would for full HTAs and that 42% utilized a methodological filter for the search (that is, to limit to only SRs and randomized controlled trials) [21]. Grant and Booth’s review of 14 RRs concluded that the completeness of the search was determined by time constraints and that restricting grey literature searching was one possible method of shortening timelines [22]. Another review found that several RRs employed only one reviewer for title/abstract screening and data extraction [15].

Ganann *et al*. found that many RRs restricted their literature searches or retrieval methods to include only readily available literature [15]. Cameron’s review found that 0% of RRs excluded SRs, 6% excluded randomized controlled trials (RCTs), 17% excluded non-RCTs, 39% excluded case series, and 83% excluded case reports [14]. The review indicated that lower levels of evidence were used in RRs if nothing else was available but that RRs focused on identifying higher levels of evidence whenever possible [14]. Grant also found that RRs limited their methods to analyse readily available review articles [14].

### Quality assessment

Brassey argued that transparency about quality assessment (QA) of the studies included in RRs was essential [6]. Scott and Harstall cautioned that a lack of QA in RRs may result in the over-representation of poorer quality research [23]. Thomas *et al*. recognized the trade-off between assessing study quality using appropriate tools without burdening the review team [17]. Harker and Kleijnen found a positive correlation between the time taken to produce the RRs and assessment of methodological quality [16] with a tendency for RRs with more robust methodology to take longer [16]. Harker and Kleijnen also found that 47% (*n* = 23) of the RRs carried out QA using a specified methodology (for example, checklist), and 29% (*n* = 14) of the RRs used some form of QA but with unclear methodology [16]. A 24% (n = 12) of RRs reported that study quality was not assessed or was simply not reported [16]. Cameron’s study found a similar estimate that 72% of RR producers conducted some form of QA, although they concluded assessments were usually brief [22].

### Data synthesis

Brassey and Cameron reported that meta-analyses are often not undertaken in RRs [6,14], and Harker and Kleijnen found that only 20% (*n* = 8) of the quantitative reviews reported a meta-analysis [16]. It is unclear if meta-analyses were feasible in the RRs examined and neglected due to time constraints or if insufficient evidence was found in the RRs on account of the topics being new. In support of their inclusion, Thomas *et al*. argued that meta-analyses were suitable for RRs owing to their ability to quantitatively summarize a range of studies [17]. Aidelsburger *et al*. also shared advice to producers of rapid health-economic HTAs that the data of included studies should be synthesized and presented in a comparable way [20]. Many authors of review articles found that narrative or thematic tabular summaries were a popular method of presenting results in RRs [14,17,19,20,22]*.*

### Rapid reviews’ conclusions/recommendations

None of the review articles described RRs including either a rating of the evidence base or a confidence measure in conclusions. Scott described a level of uncertainty in understanding how the components of a report influence its conclusions, what the minimum reporting elements would be to guarantee an accurate or reliable result, and whether these elements would vary depending on the topic of the RR [23]. One review showed that conclusions of RRs typically do not differ from SRs [6]; however, authors of many reviews agreed that the conclusions from RRs may be less generalizable or provide less certainty than standard SRs [14,15,18,23]. It is unclear, as with the question of including meta-analyses, whether a perceived lack of certainty resulted from RRs investigating new topics with a smaller evidence base or from time constraints on the review.

Reviewers concluded that RRs were not viable alternatives to SRs [15] and that decision-makers would lose the detail and assurance provided by a standard SR [24]. Grant and Booth warned that less time spent on QA or on synthesizing the evidence resulted in inconsistencies or contradictions and an over-emphasis on poor quality research [22]. While Aidelsburger *et al*. argued that conclusions of rapid health-economic HTAs should be as comprehensive as full HTAs, they recommended that producers clearly present the limitations of RR products [20]. Thomas *et al*. argued that the importance and consequences of the decision guiding the review should determine the methodological approach to the RR, with critical decisions justifying more rigorous methods resulting in increased confidence in the conclusions [17].

## Discussion

This synthesis confirms what Ganann’s SR found previously: heterogeneous RRs are being produced in the absence of standards, particularly by HTA agencies [15]. However, this synthesis of review articles about RRs allowed for the identification of common findings and conclusions that contributed to the recommendations included below.

Lacking a single definition, RRs are better understood as a spectrum of products: some use a different methodological approach compared to a standard SR, while others closely resemble a SR. Key questions pertinent to conducting reviews rapidly are the following: 1) what steps are eliminated or reduced in comparison to a standard SR and 2) what are the potential consequences of taking a different methodological approach? One review found a significant inverse association between the number of substandard or unclearly reported SR methods employed and the number of months between end search date and review publication [16]. As the timelines shrank, the number of unclear or non-standard methods used increased. The methodological approach taken influences the time needed to complete the RR, and producers need to ascertain - in consultation with the individuals commissioning the RR - how urgently the results of the RR are needed and what level of methodological rigor is acceptable given the nature of the question.

There is a perceived trade-off between time and the comprehensiveness of the end product, but the implications of cutting methodological corners to produce a rapid result are unclear. Transparency in reporting should assist clients in reaching an appropriate level of confidence in RR conclusions, and more research is needed to understand how RRs are used by decision-makers. In terms of current timelines, a single week may be sufficient to produce a RR product to meet the needs of some clients, but for a RR that approaches the rigor of a full SR, clients should be prepared to wait 6 months or more for a completed product.

Question development requires particular attention by producers, but not all reviews found that RRs consistently used well-focused clinical questions. Clients and producers should agree on a clearly articulated question that can be answered by a RR product (for example, a single-intervention effectiveness question). Standard SRs or HTAs may be more appropriate than RRs for clients seeking answers to complex questions about multiple interventions and safety, social, policy, or ethical issues.

While universally accepted search methods for RRs were not described by the review articles, many found that RRs limited the number of databases included in the search and often reduced (or eliminated) hand-searching or grey literature searching. Database search results were constrained by language and date and often used study-type filters to focus on only the highest level of evidence available (that is, SRs, guidelines, or randomized controlled trials).

Producers should consider that a less comprehensive search may find studies that confirm what is already known from the literature and that limiting results to SRs or other pre-synthesized study types may be a suitable method to ensure a rapid product. However, client questions ought to dictate search restrictions. Narrow search parameters may not yield enough results for questions that have not been extensively investigated, so for questions about recent technological innovations or new interventions, more inclusive RR search strategies may be required. Conversely, exhaustive search methods may be entirely inappropriate for new or recent interventions when scant evidence exists and clients require a shortened timeframe for completion of the RR.

There was little consistency in the way quality of included studies was assessed in RRs. A majority of authors of included review articles concluded that QA was an essential element and that failing to use a QA tool could result in contradictory conclusions. In cases where the only available evidence is poor research, QA can impact how the evidence is interpreted and used to formulate conclusions when RR authors call attention to the weaknesses of the evidence. A lack of QA may lead to over-reliance on and misinterpretation of poor research, and producers should caution clients that summarizing or analysing data from studies without considering their methodological quality may misrepresent the evidence.

Review articles found that RRs commonly included a qualitative summary of included studies but seldom conducted meta-analyses. Tables were used to compare the findings and methods of studies and may prove useful to RR clients who prefer a snapshot view of the results. Any RR analysis should make transparent the limitations of the end product based on the methodological approaches taken, particularly if no QA was conducted. Conclusions may also be less generalizable than full SRs and only applicable to the healthcare organization that commissioned the RRs.

Future research is needed to determine how, or if, the results and conclusions of RRs differ from those reached by standard SRs or how RRs differ in quality given an evidence base of comparable size. In addition, concrete guidance for conducting and reporting RRs is needed from methods experts in conducting evidence syntheses. Heterogeneous methods of production suggest that multiple products fall under the umbrella term ‘rapid review.’ Clear definitions for the range of RR products will help inform decisions to undertake a RR. The Agency for Healthcare Research and Quality (AHRQ) EPC workgroup on RRs recently developed a classification system of RR types [5] to assist producers and clients in selecting the best product for their information needs. Future research is also needed to confirm the implications for RRs of adapting their approach to produce a more rapid result.

Strengths of this analysis include the comprehensiveness of the search and the first-level screening by two independent reviewers. A limitation of this study was the use of a single reviewer for extracting and analysing review articles about RRs. Also, given current interest in rapid reviews, it was a limitation of the research that the search was not completely updated following analysis and manuscript preparation. Reviews published since November 2013, when the original search was conducted, have not been included in this analysis. Additional limitations include a potential overlap in the RRs described in each review article, the possibility that variability in RRs is a result of maturation of the review method, and the heterogeneity of review articles analysed.

## Conclusions

This analysis of review articles about RRs yielded findings that contribute to our general understanding of RR products. RR methods vary greatly, as do their definitions and applications. Strengths of RRs include the potential to answer narrow questions of efficacy or effectiveness in a shorter time and with fewer resources than standard SRs. No authors of review articles included in this analysis supported substituting SRs for any form of RR, although they recognized their unique value. Authors of RRs should always be transparent in reporting the methods used to produce the RR in a shortened timeframe and to discuss the potential limitations or perceived implications of those methods. Particular caution should be used when eliminating QA in a RR.

## Appendix

Search strategy

Search strategy for Ovid MEDLINE(R) is found in Table 1. Search strategy for Ovid EBM Reviews is found in Table 2. Search strategy for Ovid Embase is found in Table 3.

**Table 1:** **Ovid MEDLINE(R) in-process & other non-indexed citations November 6, 2013, and ovid MEDLINE (R) 1946 to October week 5 2013 (Searched: November 6, 2013)**

**Search #**	**Search command**	**Results #**
1	((rapid or mini or pragmatic or targeted or focused or brief) adj2 (((systematic or evidence or data or knowledge) adj2 (review* or synthes*)) or HTA or health technology assessment*)).ti,ab.	318
2	((rapid or pragmatic) adj2 (review* or HTA or health technology assessment* or evidence assessment*)).ti,ab.	381
3	1 or 2	656
4	3 not animals/	572
5	remove duplicates from 4	519
6	limit 5 to (english language and yr = “2000-Current”)	409

*Command in Ovid denotes unlimited right-hand truncation for word variations that are formed with different suffixes.

**Table 2:** **Ovid EBM reviews - cochrane methodology register 3rd quarter 2012 (Searched: November 7, 2013)**

**Search #**	**Search command**	**Results #**
1	((rapid or mini or pragmatic or targeted or focused or brief) adj2 (((systematic or evidence or data or knowledge) adj2 (review* or synthes*)) or HTA or health technology assessment*)).ti,ab.	22
2	(rapid adj2 review*).ti,ab.	15
3	1 or 2	32

**Table 3:** **Ovid Embase 1996 to 2015 week 08 (Searched: February 27, 2015)**

**Search #**	**Search command**	**Results #**
1	((rapid or mini or pragmatic or targeted or focused or brief) adj2 (((systematic or evidence or data or knowledge) adj2 (review* or synthes*)) or HTA or health technology assessment*)).ti,ab.	439
2	((rapid or pragmatic) adj2 (review* or HTA or health technology assessment* or evidence assessment*)).ti,ab.	512
3	1 or 2	877
4	3 not animals/	857
5	remove duplicates from 4	826
6	limit 5 to (english language and yr = “2000-Current”)	734
7	limit 6 to (exclude medline journals and embase)	63

SRC Methods Library

Searched: October 21, 2013

Descriptor search: systematic reviews - rapid

27 records retrieved

## Additional file

Additional file 1:
**Results table.** Results table includes findings and recommendations from review articles analyzed in the manuscript: reference information for analyzed reviews, number of RRs analyzed, RR definitions provided in the text, timelines for RRs included, RR scope and question types, RR search strategy restrictions, RR study quality assessment, RR data synthesis, and RR conclusions.

## Abbreviations

AHRQ: Agency for Healthcare Research and Quality
CADTH: Canadian Agency for Drugs and Technologies in Health
EPC: Evidence-based Practice Centers Program
HTA: health technology assessment
QA: quality assessment
RCTs: randomized controlled trials
RR: rapid review product
SR: systematic review
SRC: Scientific Resource Center of the Evidence-Based Practice Centers Program
