# Supplementary material for: Advancing knowledge of rapid reviews: an analysis of results, conclusions and recommendations from published review articles examining rapid reviews
Source: Syst Rev. 2015 Apr 17;4:50. doi: 10.1186/s13643-015-0040-4 (PMC4415284; doi:10.1186/s13643-015-0040-4)
Supplement: Additional file 1: — Results table. Results table includes findings and recommendations from review articles analyzed in the manuscript: reference information for analyzed reviews, number of RRs analyzed, RR definitions provided in the text, timelines for RRs included, RR scope and question types, RR search strategy restrictions, RR study quality assessment, RR data synthesis, and RR conclusions. [file 13643_2015_40_MOESM1_ESM.docx]

Additional File 1: Findings and recommendations table from included review articles

| **Author,year  [reference]** | **# of RRs** | **RR definition** | **RR  timeline** | **RR scope and question types** | **RR search strategy restrictions** | **RR study quality assessment** | **RR data synthesis** | **RR conclusions** |
| --- | --- | --- | --- | --- | --- | --- | --- | --- |
| Abrami 2010  [19] | 24 | “There seems to be little consistency in terminology and methodology with respect to what we have called brief reviews.” | Several  days to  1 year | Careful focusing of the question can help to make the product more rapid. | More likely to exclude studies of lower quality | Authors should use a simple quality appraisal tool. | Typically narrative and tabular. | Increased possibility for bias compared to full SRs. |
| Aidelsburger 2003  [20] | 3 | “A rapid health-economic HTA is a unique method for a targeted assessment of the cost-effectiveness of a medical technology within a short time frame.” | 3-6 mo. | Study question should reflect the interests of the recipient. Clear definition will help limit scope. | Should not restrict search. | Authors should assess study quality. | Decision analysis may be appropriate. | Discussion, conclusion, and recommendations should be as conclusive and comprehensive as those in full SRs. |
| Brassey 2012  [5] | 10 | “The notion of a rapid-review is ill-defined.” | < 5 wk. | Appropriate: Efficacy and effectiveness. Inappropriate: Safety, economics, and ethics. | NR | Authors should assess study quality. Best RRs are transparent about quality assessment. | Meta-analysis often not undertaken. | Do not typically differ from conclusions of full SRs. |
| Cameron 2007  [14] | 36 | “Any HTA report or SR that has taken between 1-6 months to produce which contains the elements of a comprehensive literature search.” | 1-6 mo. | Appropriate:  Efficacy and safety. Inappropriate: Economic and ethics. | Can restrict number of databases searched, gray literature search, and hand-searching. | Quality assessment was conducted in 72% of RR products examined. | Narrative summary. Meta-analysis often not undertaken. | Can state general conclusion of RR, but avoid quantifying or providing detailed discussions. |
| Dennett 2012  [21] | 32 | “An HTA report where methodological compromises are made in order to meet shorter timelines.” | 1 wk. to  6 mo. | NR | Can restrict number of databases searched; can also use more precise search strategies and methodological filters. | NR | NR | NR |
| Ganann 2010  [15] | *70 | “[Wide variation] in language used, timeframes, content, and methods.” | 3 wk. to  6 mo. | Appropriate: Efficacy and effectiveness. Inappropriate: Economics and complex questions. | Can restrict number of databases searched, gray literature search, and language and year of publication. | Authors should assess study quality. Priority should be placed on quality assessments over extensive literature searching. | NR | May be less generalizable and provide less certainty than full SRs. |
| Grant 2009  [22] | 14 | “Assessment of what is already known about a policy or practice, by using SR methods to critically appraise existing research.” | NR | NR | Can restrict gray literature search; can also use more precise search strategies. | Authors should use a simple quality appraisal tool. | Typically narrative and tabular summaries. | NR |
| Hailey 2009  [24] | †15 | “Rapid HTAs fall within a continuum of assessment products, somewhere between “full HTAs” with a rigorous approach at all stages, and mini-HTAs or horizon scanning reports.” | 1-6 mo. | Appropriate:  Health technology (specifically emerging technologies). | NR | NR | NR | Trade-off between providing relatively rapid advice to decision makers and losing detail and assurance provided by a more comprehensive process. |
| Harker 2012  [16] | 46 | “[No] clear or final definition of what a RR is and how the methodology differs from a full SR. The term ‘RR’ does not appear to have one single definition but is framed in the literature as utilising various stipulated time frames between 1 and 6 months.” | §7-12  mo. | Appropriate: Efficacy and effectiveness. | Can restrict number of databases searched. | RRs with more robust methods for quality assessment often take longer to produce. | Meta-analysis often not undertaken. | NR |
| Scott 2012  [23] | ‡3 | “There is no universally agreed upon description or methodology for RR products. These products [have] heterogeneous timelines, components, search strategies, and methodologies, which reflects a common imperative  [to address] the needs of policy makers.” | <1 mo.  to >6 mo. | Inappropriate: Safety, economics, ethics, questions requiring interdisciplinary or external input. | Limitations depend on product type, but may also be product dependent. | Lack of quality assessment may cause results from poorer quality research to be over-represented. | Data analysis depends on product type. | The effect of methodology on the conclusions of RRs is currently unclear. |
| Thomas 2013  [17] | 2 | “REAs are expected to perform much the same function as full SRs and conform to many of the same standards – but in a shorter timescale and usually with a lower budget.” | 3 mo. | Appropriate: Efficacy and effectiveness. Inappropriate:  Social policy. | Determined by topic | Authors should quickly assess study quality. | Thematic summaries and meta-analysis. | As a general rule, the more important it is that confidence in the validity of the findings is required, then the more careful we need to be about the limits placed on the RR. |
| Watt 2011  [18] | 7 | “[Be] produced within 1–6 months, contain elements of a comprehensive or systematic/quasi-systematic search, [be] produced in full text by a not-for-profit organization and [be] published between 2001 and 2004... RR products produced by HTA agencies are not well defined and [have] highly variable [methods].” | 1-6 mo. | Appropriate: Efficacy and effectiveness. Inappropriate: Safety, economics, and ethics. | Can restrict search (methods not specified). | NR | NR | The essential conclusions of the rapid and full reviews did not differ extensively across the topics. |

HTA: health technology assessment; NR: not reported; REA: rapid evidence assessment; RR: rapid review; SR: systematic review.

*–45 methodological articles, 25 RRs

†–rapid HTA

‡–product inventories

§–3 months or less to 19-24 months
